# American Medical Association—Session for 1869

**Published:** 1869-06-01

**Authors:** 


### CHICAGO MEDICAL JOURNAL.

Vol. XXVI.—JUNE 1, 1869.—No. 11.

### REPORTS OF MEDICAL SOCIETIES.

### *American Medical Association—Annual Session for 1869.*

[We are indebted to Prof. E. S. Gaillard, of the *Richmond & Louisville Medical Journal*, for the appended succinct account of the doings of the "The Medical Congress" at New Orleans.]

LIST OF OFFICERS OF THE ASSOCIATION.

The following is the list of officers of the Association for 1869:

President—Wm. O. Baldwin, M. D., Montgomery, Ala.

Vice Presidents—George Mendenhall, M. D., Cincinnati, O.; Noble Young, M. D., Washington D. C.; N. P. Moore, M. D., Portland, Maine; S. M. Bemiss, M. D., New Orleans, La.

Permanent Secretary—Wm. B. Atkinson, M. D., Philadelphia.

Assistant Secretary—A. J. Semmes, M. D., Savannah, Ga.

Treasurer—Caspar Wistar, M. D., Philadelphia.

The officers of the Association are elected annually, on the recommendation of a nominating committee, composed of one from each State delegation represented, and one from the Medical Staff of the Army and Navy. The individuals so recommended are, on motion, elected by the Association before each annual adjournment. The officers of the present year were elected at the meeting of the Association held last May in Washington City.

PROCEEDINGS OF THE OPENING DAY.

The American Medical Association met in the Mechanics' Institute, at 11 A. M.

The President, Dr. W. O. Baldwin, of Alabama, occupied the chair, assisted by Vice Presidents Drs. George Mendenhall, of Ohio, and S. M. Bemiss, of Louisiana.

The Permanent Secretary, Dr. W. B. Atkinson, of Pennsylvania, and Assistant Secretary, Dr. A. J. Semmes, of Georgia, were present.

The President invited to seats on the platform Drs. Warren Stone and A. Lopez, of New Orleans, and ex-Presidents H. F. Askew, of Delaware, N. S. Davis, of Illinois, and Alden March, of New York.

The session was opened with prayer by Rev. Mr. Gallaher, of New Orleans.

Dr. T. G. Richardson, of Louisiana, chairman of the Committee of Arrangements, welcomed the delegates to the city in an eloquent address.

The President then delivered the annual address.

Letters were read by the Permanent Secretary from Drs. S. D. Gross, of Pennsylvania, W. Byard and W. Canniff, of Canada, and R. A. Kinloch, chairman of the Medical Society of South Carolina, expressing regret at their inability to be present on this occasion.

On motion of Dr. Mussey, it was resolved that each State Medical Society be requesed to prepare an Annual Register of all the regular practitioners of medicine in their respective States, giving the name of the colleges in which they may have graduated, and date of diploma or license.

Special reports of committees were then made as follows:

On Devising a Plan for the Relief of the Widows and Orphans of Medical Men, Dr. H. Griscom, New York, chairman, reported, which was referred to the Committee on Publication.

On Veterinary Colleges, Dr. Thomas Antisell, District of Columbia, chairman, reported progress and was continued.

On Specialities in Medicine, and the Propriety of Specialists Advertising, Dr. E. Lloyd Howard, Maryland, chairman, reported and made the special order for Wednesday at 12 M.

On Library of American Medical Works, Dr. J. M. Toner, District of Columbia, chairman, reported, and was, on motion of Dr. Davis, made special order for Wednesday at 1 P. M.

On the Best Method of Treatment for the DifferentForms of Cleft Palate,—Dr. J. R. Whitehead, N. Y., chairman. Reported and referred to section on Surgery.

On Rank of Medical Men in the Navy—Dr. N. S. Davis, Illinois, chairman, announced that their last year's report was final, and committee was discharged.

The report on Medical Ethics by Dr. D. Francis Condie, Pennsylvania, chairman, was read by Dr. Davis and adopted.

On American Medical Necrology—Dr. C. C. Cox, Maryland, chairman, reported progress, and was continued on motion of Dr. Davis. Dr. Cox was authorized to fill all vacancies on his committee.

Voluntary communications were presented by Dr. Jos. Tones, of Louisiana, on Mollities Ossium, and referred to section on Surgery.

A. Sayre. Referred to section on Hygiene. On Cases of Lead Palsy from use of Cosmetics, by Dr.. L.

On the Physiology and Chemistry of Longevity, by Dr. Cutler, of Mississippi. Referred to section on Hygiene.

On the Protective and Preventive uses of Quinine, by Dr. S. Rogers, of New York. Referred to section on practical Medicine.

On the Tongue in Malarious Diseases, by Dr. Osborn, of Alabama. Referred to section on Practical Medicine.

On the Warm Cerebro-spinal Bath in the Treatment of congenital apnœa, and on a newmethod of artificial respiration, by E. D. McDaniel, of Alabama. Referred to section on Practical Medicine.

Reports on Climatology and Epidemics were received from Drs. Thomas, of New York; T. J. Heard, of Texas; F. W. Hatch, of California; E. A. Hildreth, of West Virginia, which were referred to the section on Climatology and epidemics.

On motion of Dr. Davis, the report on the revision of the plan of organization was made the special order for Wednesday, at 10 A. M.

Near the hour of adjournment to-day Dr. Eve suggested that the many papers on education, ready to be offered, be laid before the Association on the next morning at 9 o'clock.

Dr. Davis, of Chicago, moved that all such papers be referred to a special committee of five.

Dr. Eve seconded this motion, which being carried, the President appointed Drs. Davis, of Illinois; P. F. Eve, of Tennessee; E. S. Gaillard, of Kentucky; E. Lee Jones, of New York; and J. K. Bartlett, of Wisconsin.

On motion adjourned until Wednesday at 9 A. M.

The special feature of this day's proceedings was the delivery of the President's address, which was universally commended and admired.

SECOND DAY'S PROCEEDINGS.

The attendance yesterday was much larger than on the first day. A number of arrivals had increased the *person-*

*nel* of the Association to near three hundred. The hall was well filled, and the same dignified deportment which had marked the deliberations of the members on the opening day was displayed on the second day. Much of the facility with which the deliberations were conducted yesterday, and the promptness with which action was taken upon the various resolutions, was, undoubtedly, due to the tact and practical administrative knowlege displayed by President Baldwin.

At 9 A. M. Dr. W. O. Baldwin, the President, in the chair, called the meeting to order.

A paper on "Canula and the New Mode of Applying Ligatures" was submitted by Dr. P. F. Eve (Tenn.), and was referred to the Section on Surgery.

Dr. J. M. Bush, of Kentucky, offered the following resolution:

*Resolved*, That a committee of five members be appointed by the chair, to take into consideration the subjects alluded to in the President's address, and report at this meeting.

This resolution having been adopted, the President selected as members of the committee Dr. Parvin, of Indiana, chairman; Dr. Toner, of the District of Columbia; Dr. Pollock, of Pennsylvania; Dr. Welch, of Texas; Dr. Seely, of Alabama.

Dr. McPheeters, of Missouri, offered a communication from the Medical Association of that State, in reference to medical education.

On motion of Dr. Toner, District of Columbia, it was referred to the special committee on that subject.

Dr. Eve offered the minutes of the Medical Society of Tennessee, which was similarly referred.

Dr. Gaillard, of Kentucky, offered the following preamble and resolutions, which were refered to the same committee:

WHEREAS, The medical teachers of America have, after a trial of twenty-two years, failed to meet satisfactorily and efficiently the requirements of the great body of the profession in regard to medical education; and

WHEREAS, The condition of the profession is yearly becoming more deplorable on account of the antagonistic and objectionable policy of medical schools, in making the amount of fees charged, rather than scientific teaching, the basis of competition; and

WHEREAS, To obtain professionally competent graduates, sound and efficient teachers are indispensably necessary; and

WHEREAS, Such teachers, to be found throughout the country, can not be induced to leave their homes without the assurance of competent remuneration; and

WHEREAS, Such remuneration can only be obtained by adequate fees charged, unless by a system of low fees the number of students be relied upon to make up the inevitable pecuniary deficiency; and

WHEREAS, Reliance upon numbers of students for this purpose deplorably crowds the already overcrowded professional field, diminishing thereby individual income, judgment, experience, and skill, thereby compelling practitioners to resort to other avocations as a source of supplemental income; and

WHEREAS, This devotion to other pursuits destroys opportunities for study and improvement, degrading thereby the status and standard of American physicians; and

WHEREAS, The schools of New England, New York, Pennsylvania, Maryland, Virginia, South Carolina, Georgia, Missouri, Tennessee, Louisiana, Alabama, and District of Columbia now charge comparatively remunerative fees; and

WHEREAS, The *low system* of fees is charged only in a few of the Middle States, and can with advantage be made to conform to the rate of fees charged elsewhere; and

WHEREAS, It is as unethical for colleges to underbid each other pecuniarily as it is for practitioners to do so;

*Resolved*, That hereafter no medical school in this country, other than those fully endowed, be entitled to representation in this Association, if the amount charged by such schools for a single course of regular lectures be less than one hundred and forty dollars.

*Resolved*, That all schools charging less than this sum are earnestly requesed by this association to advance their rates of fees to the amount mentioned.

The report of Dr. Lee, of New York, the delegate to the Association of Superintendents of Insane Asylums, was offered and referred to the Section of Psychology.

The report of Dr. Gross, of Pennsylvania, delegate to the Foreign Medical Association, was presented, together with the letter to Dr. Ehrenberg, was read and referred to the Committee of Publication.

The time having arrived for consideration of revision of plan of organization, it was, on motion, taken up.

A recess was taken to allow the selection of members of the Committee on Nominations.

On re-assembling, the permanent Secretary announced the following as the Committee on Nominations:

New York—J. C. Smith.
Delaware—H. F. Askew.
Pennsylvania—A. M. Pollock.
Kentucky—H. M. Skillman.
Tennessee—J. B. Lindsley.
Mississippi—W. Y. Gadbury.
Alabama—J. Cochran.
Ohio—Jno. Townsend.
Indiana—B. S. Woodworth.
Illinois—T. D. Fitch.
Wisconsin—H. Van Dusen.
Missouri—J. S. Moore.
Michigan—J. B. White.
Georgia—R. D. Arnold.
Louisiana—S. Logan.
Texas—S. M. Welch.
Minnesota—C. N. Hewes.
Arkansas—R. G. Jenning.
West Virginia—W. J. Bates.
Rhode Island—G. L. Collins.

District of Columbia—L. W. Ritchie.

United States Army—J. J. Woodward.

United States Navy—F. E. Potter.

Dr. Chaille, of Louisiana, submitted a proposition for a common medical nomenclature in the United States, taking as a model an official publication on the subject by the Royal College of Physicians, London, and offered the following resolutions, which were adopted:

*Resolved*, That a committee of five be appointed by the President, to report as soon as practicable to the present session of this association upon the following:

1. The propriety of adopting, and using its influence to have adopted by the entire medical profession in the United States, the provisional "Nomenclature of Diseases of the Royal College of Physicians."

2. On the practicability of having this nomenclature published in such manner as may render it easily and cheaply accessible to every member of the profession.

3. To recommend such other practical measures for the action of this association as may be necessary to introduce this nomenclature into official (military, naval, etc.) and general use.

The Chair appointed the following gentlemen as the committee: Drs. Woodward, U. S. A.; Houstis, of Alabama; F. G. Smith, of Pennsylvania, and Chaille, of Louisiana.

The reports of the Committee of Publication, and the Treasurer, were read, accepted, and referred to the Committee of Publication.

On motion, the Committee on Nominations were permitted to retire for consultation.

The special order for 12 being the report on Specialists, it was read by the Secretary, and, on motion of Dr. Sayre, the resolutions were adopted and the report referred to the Committee of Publication.

The large number of arrivals has increased the *personnel* of the Association to near four hundred.

Dr. L. P. Yandell, Jr., of Kentucky, introduced the following resolution:

*Resolved*, That private handbills addressed to the members of the medical profession, or advertisements in newspapers, calling the attention of professional brethren to themselves as specialists, be declared in violation of article — of section — of the Code of Ethics of the American Medical Association.

Dr. N. S. Davis, of Illinois, said it had been the practice to publish cards in medical journals for the purpose of informing the medical fraternity that the advertiser devotes himself to special diseases. These cards were not so much for the information of the public as for the medical fraternity. He hoped that, now the question was up, it would be discussed fully.

Dr. L. P. Yandell, of Kentucky—We have allowed physicians to violate the code of ethics by advertising in medical journals that they are specialists in the treatment of certain diseases. In Europe they are stricter in regard to specialists than here. There, where a physician wins a reputation in the treatment of certain diseases, his professional brethren send cases to him for treatment, but advertisement is prohibited. If we are allowed to resort to advertisements, not as a question of merit, but of money, this association should so declare. I am sure I am right in principle, and I want to get an expression from this association.

Dr. Sayre, of New York—Let those who understand the best mode of treatment in special diseases instruct their professional brethren through the proper channels, as the honorable way of preferment, not by advertising as a matter of dollars and cents. Let us look the matter square in the face, and sustain the resolution of Dr. Yandell. May my hand be paralyzed if I make any attempt to profit by advertising the knowledge I have attained in my profession.

Dr. Mussey, of Ohio, moved to amend by inserting, "or in medical journals."

This amendment was accepted.

Dr. Davis, of Illinois—The question before us is one to settle the interpretation of the existing statute. The question is whether that rule of ethics shall be enforced, prohibiting publication of cards calling attention of individuals laboring under particular diseases.

Dr. Yandell stated that he had preferred charges against a practitioner of Louisville for advertising himself as a specialist in the newspapers, and for sending handbills to physicians, but that the medical societies of that city not having sustained him, he brought this subject up for the action of this association. The question is, shall we associate with professional prostitutes and medical outlaws?

Dr. Yandell's resolution was unanimously adopted.

The Committee on Prize Essays reported as follows—their report being adopted:

They have received but two essays—one upon "The Physiological Effects and Therapeutical Uses of Atropia and its Salts;" the other upon "Quinine as a Therapeutic agent." They agree to present both of these essays to the association, and to recommend the award of a prize of one hundred dollars to each of them.

S. M. Bemiss, *Chairman.*
C. Beard,
Joseph T. Scott,
S. A. Smith.

The Secretary broke the seals, and announced that Dr. S. S. Herrick, of New Orleans, was the author of the paper on Quinine, and Dr. Roberts Bartholow, of Cincinnati, was the author of that on Atropia.

Dr. Booth, of Mississippi, ofiered the following preamble and resolution:

*Resolved,* That the proper construction of art. 4, sec. 1, Code of Ethics, A. M. A., having been called for, relative to consultation with irregular practitioners who are graduates of regular schools.

*Resolved,* That said art. 4, sec. 1, Code of Ethics, A. M. A., excludes all such practitioners from recognition by the regular profession.

This resolution was unanimously adopted.

On motion, the Association adjourned until Thursday, at 9 A. M.

THIRD DAY, THURSDAY, MAY 6, 1869.

The proceedings of yesterday were undoubtedly the most important held since the opening of the session. The reports of special committees—some of them bearing upon questions vital to the interest of medical science—were read and appropriately acted upon. Among the ablest reports was that on President Baldwin's address, and one from the Committee on Medical Education. We give the former; but we regret that the great length of the latter prevents us from publishing, at this time, even a summary. We should judge that much was done yesterday toward defining clearly the position to be assumed during the coming year by the association. Many of the technical reports are of a high order of ability.

Dr. Baldwin, President, in the chair; Dr. S. M. Bemiss and Dr. Mendenhall, Vice Presidents.

Reading of the minutes was dispensed with.

On Dr. Parvin's report of Committee on President's Address, your committee desire to present the following report:

"We cannot refrain, before entering upon the consideration of the plan recommended by the President for the improvement of medical education, gladly expressing our high appreciation of the general tone of this address, of the broad and catholic spirit which pervades it, finding expression in earnest and eloquent words—in brief, we believe the address worthy the perusal of every member of the profession, in that it was worthy the memorable occasion, and is worthy of a place in the annals of medicine.

"On the other hand, we cannot refrain, with sadness be it said, from acknowledging the truth of the terrible allegations made against the present condition of medical education, and the little success attending the efforts for improvements in such connection, made during a score of years.

"The special recommendation made by the President is in these words:

"'I would advise that we appoint a committee of our wisest and best men to digest a plan for one or more National Medical Schools, and to memorialize Congress in behalf of the enterprise. Let the plan embrace as a basis the features presented by the Cincinnati Convention of Teachers; let these schools or universities confer such distinctions and privileges as will be proportionate to the superiority they demand, and such as will make the attainment of their diploma an object of the ambition of those who engage in the study of medicine; let the choice be open to all aspirants, and the appointment or election of professors so guarded as to secure the very highest talent, the most profound learning, with the most fully demonstrated capacity for teaching. Make the salaries of the professors large, and not to depend upon the number of students; and let the Federal Government assume a proper share of the expenses incurred.'

Your committee express their hearty approval of this general plan, but suggest that the effort at first should be for the establishment of but a single school, as more feasible, and beside, one such institution would be a model which other medical colleges might in time be induced to imitate in extent, duration and thoroughness of teaching, in rigidness of requirements for the degree of M. D.

"We likewise desire to say that when the details of this general plan are thrown into form, there should be the amplest security against the places and the power of such a medical college as designed, ever falling into the hands of politicians or the proteges of politicians. Medicine is higher than politics; broader than political creeds and party platforms.

"In conclusion, your committee reiterate the recommendation of the President as to the appointment of a committee for the special purposes referred to."

The President appointed this committee as follows: Dr. N. S. Davis, of Chicago; Dr. F. Gurney Smith, of Philadelphia; Dr. D. H. Storer, of Boston; Dr. E. S. Gaillard, of Louisville; Dr. Joseph Jones, of New Orleans.

On motion, Dr. W. O. Baldwin, of Montgomery, was added to the committee.

The President appointed as delegates to the British Medical Association:

Dr. N. Pinckney, U. S. N., R. R. McIlvain, Ohio, J. F. Hibberd, Indiana, B. Lindsey, D. C. G. C. Blackman, Ohio.

To the Canadian Association:

Dr. Alden March, Albany, N. Y.

Dr. Davis read report of meeting of editors, and presented the following from the Association of American Medical Editors:

*To the American Medical Association:* I have been instructed to announce to your honorable body, that those members of your association in attendance at this annual meeting, after proper consultation, have effected a permanent organization with the title of "The Association of American Medical Editors." The objects of this organization are the cultivation of friendly relations; mutual assistance; community of effort and views, where possible, in a system of receiving foreign exchanges, and sending our own journals abroad; concert of action in support of improvement in the present system of medical education, and of a higher standard of preliminary attainments for those who propose to enter upon the study of medicine; in proposing laws for the proper registration of births, marriages and deaths; in collecting the names of all the regular practitioners in the several States; and in promoting generally the value and efficiency of our periodical medical literature. The association thus formed is to hold its annual sessions on the day preceding the annual meetings of this body, and in the same localities. Dr. Mitchell, of New Orleans, is the Permanent Secretary, and Dr. J. B. Lindsley, of Nashville, Tenn., the Assistant Secretary. Congratulating your honorable body on the establishment of another organized power within the ranks of your noble profession,

I remain yours, very truly,

N. S. DAVIS, Editor,

*President of Association of American Medical Editors.*

Referred to Committee on Publication.

In the case of a special violation of the code in Louisville, Kentucky, Dr. Gaillard desired to remove an unfortunate and incorrect impression created in the minds of the members of the Association by one of the delegates from Louisville (Dr. Yandell), in regard to a failure on the part of two medical societies in that city to meet promptly and fully and alleged breach of the code of ethics on the part of one of the members of these societies.

Dr. Gaillard stated that he was satisfied Dr. Yandell had no intention of creating a false impression in this connection. The gentleman against whom charges had been preferred was a German of professional proficiency, who recently arrived, and, ignorant of the code of ethics, had advertised himself as a specialist in the daily papers, and had sent private handbills to professional men. As soon as this gentleman was apprised of his fault, he had promptly withdrawn his advertisement from the daily papers and had ceased sending his handbills mentioned. The medical societies of Louisville decided that though there had been a breach in the letter of the code of ethics, the gentleman arraigned had no intention of doing what was professionally wrong. These societies, therefore, declined to expell the member against whom these charges had been preferred. Dr. Gaillard thought it due to those societies and to the gentleman offending, that this explanation should go upon the records.

[CONCLUDED NEXT MONTH.]

